# Epilepsy, Antiseizure Therapy, and Sleep Cycle Parameters

**DOI:** 10.1155/2013/670682

**Published:** 2013-08-07

**Authors:** Vladimir Shvarts, Steve Chung

**Affiliations:** Department of Neurology, Barrow Neurological Institute, 500 West Thomas Road, Suite 720, Phoenix, AZ 85013, USA

## Abstract

A reciprocal relationship exists between sleep and epilepsy. The quality of sleep is affected by the presence and frequency of seizures, type of antiepileptic therapy utilized, and coexisting primary sleep disorders. Daytime somnolence is one of the most common adverse effects of antiepileptic therapy, with specific pharmacologic agents exhibiting a unique influence on components of sleep architecture. The newer generation of antiseizure drugs demonstrates improved sleep efficiency, greater stabilization of sleep architecture, prolongation of REM sleep duration, and increased quality of life measures. The emerging field of chronoepileptology explores the relationship between seizures and circadian rhythms, aiming for targeted use of antiseizure therapies to maximize therapeutic effects and minimize the adverse events experienced by the patients.

## 1. Introduction

Although the complex relationship between sleep and epilepsy has not been fully elucidated, it is well known that sleep disturbance provokes seizures and that seizure activity may influence the quality of sleep. In addition, antiepileptic drugs (AEDs) that are commonly used for seizure treatment affect sleep quality and architecture. Some AEDs tend to cause sleepiness or drowsiness while others can lead to insomnia. Sleep is an essential physiologic state that influences restorative and memory consolidating functions [1]. As previously recognized, the relationship between epilepsy and sleep disturbance is likely multifactorial: the direct effect of seizures, adverse events due to AED therapy, presence of psychiatric comorbidity, and coexisting sleep disorders all have the potential to contribute to alteration of sleep architecture and the subjective quality of sleep. Accordingly, one would expect that lack of sound sleep would significantly impact neurocognitive and psychological function, especially in patients treated with AEDs for their seizures. It is important for clinicians to understand the proclivity of a specific AED to affect the quality of sleep in order to guide epilepsy therapy and prevent disturbance of a patients' nocturnal recovery. This review systematically evaluates the currently available literature, elucidating the effect of antiepileptic drug therapy upon the sleep cycle. A search of relevant primary research and review articles was performed utilizing the PubMed database.

## 2. Epilepsy and Sleep

Sleep is classically divided into REM and non-REM phases as defined by the parameters of electroencephalography, respiration, eye movement, and electromyography. The non-REM phase consists of the light stages of sleep—N1 and N2 (previously designated stages 1 and 2), followed by deeper predominantly slow wave sleep (SWS)—N3 (previously divided into stages 3 and 4). Disturbance of sleep is consistently ranked among the top three adverse side effects in patients with epilepsy. Subjective sleep complaints in the prior 6 months were reported by up to 39% of patients with partial epilepsy as compared to 18% of controls [2]. The largest differences were observed in measures of excessive daytime sleepiness (13.8%) and psychiatric sleep disorder (14.1%). The authors concluded that sleep disturbance contributes to a lower quality of life independent of epilepsy diagnosis or its treatment. Sleep complaints in adult patients with epilepsy reported in other questionnaire-based studies varied from 16.9% to 36% [3]. 

Both NREM sleep and its deficit promote epileptiform discharges, with more profound effect on diffuse discharges. In contrast, during REM sleep, the topology, distribution, and frequency of epileptiform discharges are decreased [4]. A number of authors attribute facilitation of epileptiform activity during NREM sleep to increased synchronization of the EEG pattern; in contrast, inhibition of epileptiform activity during REM sleep could be explained by desynchronization of cerebral networks [3].

Sleep efficiency is the time spent asleep divided by the time spent awake during a given sleep period. In normal subjects this value should be more than 90% [1]. Frequent clinical and subclinical arousals as well as changes in the amount of time spent in a particular stage of sleep independent of nocturnal seizures or AED use have been described in patients with epilepsy [5]. Frequent phase shifts increase the frequency of seizures and interictal epileptiform discharges [6]. In turn, both nocturnal and daytime complex partial seizures fragment sleep due to frequent awakenings, an increase in the number of stage shifts, a reduction in the duration of REM and slow wave sleep, and prolonged sleep onset and REM latency [7–11]. In patients with temporal lobe complex partial seizures, nocturnal seizures reduce sleep efficiency, decrease stages 2 and 4 sleep, and increase stage 1 sleep [11]. 

Drowsiness or daytime somnolence seems a more prominent adverse effect of older AEDs with a greater incidence of somnolence in patients on combinations of antiepileptic agents [12]. Rapid escalation of daily dosing is an additional factor predisposing to a greater chance of reporting a decreased daytime vigilance. As a rule, tolerance develops shortly after initiation of therapy; however, in some patients the complaint of drowsiness might be persistent and, occasionally, may lead to poor compliance or discontinuation of the prescribed agent. The Multiple Sleep Latency Test (MSLT) and Maintenance Wakefulness Test (MWT) are the most widely used objective measures of degree of daytime somnolence [12–14] that allow quantification and refinement of subjective reports of sleep disturbances in patients with epilepsy [13]. The clinical role of other electrophysiological modalities such as quantitative EEG is still to be determined [15].

Insomnia is defined by DSM-IV as difficulty initiating or maintaining sleep, nonrestorative sleep, and significant impairment in daytime functioning for at least 1 month in duration. Research criteria defines insomnia as sleep latency of more than 30 minutes, sleep efficiency of less than 85%, and sleep disturbance occurring more than 3 times per week [16]. The prevalence of chronic or severe insomnia in the general population has been estimated to approach 10% [17]. Vendrame et al. utilized a questionnaire-based survey of 152 patients with the diagnosis of epilepsy. The prevalence of moderate and severe insomnia amongst this group was 51%, with a stronger association in patients on a higher number of AEDs and coexisting depressive symptoms. Both sleep quality and insomnia were significant predictors of lower quality of life. Insomnia did not correlate with worse seizure control. A potential limitation of the study was a lack of control for the presence of undiagnosed sleep apnea [18]. Other authors reported similar insomnia rates (50% and 52%) in adult patients with a diagnosis of epilepsy [3]. Reported rates of insomnia in epilepsy patients for specific AEDs are 2.2% (CBZ), 4.9% to 6.4% (LTG), 4.2% to 6.3% (LEV), 5% (PGB), 2.3% (TPM), 3.4% (VPA), and 6.6% (VGB) [19]. 

## 3. Effect of AEDs on Sleep Architecture

First-generation AEDs (barbiturates, benzodiazepines, phenytoin, and phenobarbital) tend to reduce the amount of time spent in REM and slow wave sleep. They also tend to fragment nighttime sleep by increasing the number of arousals and stage shifts while promoting daytime sleepiness [9, 20]. These agents tend to reduce the length of time spent in REM sleep, which is implicated in learning, memory processing, and brain plasticity. The proportion of REM sleep is increased in childhood and after intensive cognitive tasks; its prolongation and improved quality positively influence daytime cognitive performance. Sleep consolidation observed with the newer generation of AEDs may contribute to reduction of seizure threshold in susceptible patients [5]. The use of AEDs without detrimental effect on “essential sleep” may lead to increased quality of life measures [10]. Effects of particular AEDs on specific parameters of MSLT are summarized in Table 1.

### 3.1. Phenytoin (PHT)

PHT inhibits voltage-gated use-dependent sodium channels. Subjective somnolence, sedation, or sleep disturbance have been reported in 23.1% of patients with epilepsy on PHT therapy [19]. PHT leads to a reduction in sleep efficiency, a decrease in sleep latency, and shortening of light sleep stages (N1 and N2) as well as the REM phase of the sleep cycle. Slow wave sleep increases in duration or does not change. Acute effects of PHT administration include a decrease in sleep-onset latency and the light stages of sleep with concomitant increase in slow wave sleep (SWS). The chronic use of PHT leads to an increase in the duration of light stages of sleep and a decrease in SWS [5, 6, 10, 21].

### 3.2. Phenobarbital (PBT)

PBT binds to the GABA_A_ receptor, enhancing the influx of chloride ions. It reduces the number of awakenings and sleep latency while increasing the length of NREM sleep and reducing REM phase duration [5, 6, 10].

### 3.3. Valproic Acid (VPA)

VPA inhibits GABA degradation, blocks voltage-gated sodium channels, and reduces calcium currents. Subjective somnolence has been reported in 2.3% to 45% of patients with epilepsy on VPA therapy [19]. Early studies in patients on VPA therapy reported no effects on sleep architecture with stabilization of sleep cycles. Other studies suggested that VPA increases the number of arousals, prolongs the light stages of sleep and NREM phase, and decreases the length of the REM phase [5, 6, 10].

### 3.4. Carbamazepine (CBZ)

The anticonvulsant effect of CBZ is mediated through inhibition of voltage-gated sodium channels. Subjective somnolence has been reported in 22% to 32.3% of patients with epilepsy on CBZ therapy [19]. Gigli et al. compared subjective and objective indices of daytime somnolence in patients with newly-diagnosed temporal lobe epilepsy with results from a group of healthy volunteers. Subjects from both groups were on 400 mg twice a day dosing of CBZ for a duration of one month. Polysomnography was performed to assess changes in sleep architecture. The initial administration of CBZ leads to an increase in the number of sleep stage shifts, a reduction in REM sleep, increased fragmentation of REM sleep, and a significant reduction in sleep latency. Upon completion of the 1-month trial period the aforementioned effects on sleep architecture were reversed, with values not significantly different from baseline measures [5, 6, 10, 21, 22]. An increase in slow wave sleep duration and sleep efficiency were the significant finding when Cho et al. evaluated 15 patients with partial epilepsy on 400 mg/day of CBZ-CR [23].

### 3.5. Ethosuximide (ETX)

ETX selectively inhibits T-type calcium channels in thalamic neurons. It has been reported to increase REM sleep phase, reduce SWS, enhance the light stages of sleep, and increase the number of awakenings after sleep onset (cited in [6, 10]). 

### 3.6. Benzodiazepines (BDZ)

The antiepileptic mechanism of action of BDZs is associated with binding to GABA_A_ chloride channels, which contribute to increased neuronal inhibition. Subjective somnolence has been reported in 25% to 33.3% of patients with epilepsy on diazepam therapy [19]. The use of benzodiazepines has classically been associated with enhanced sleep-onset latency, increased length of the light stages of sleep, decreased SWS, prolonged REM sleep latency, and reduced overall REM sleep duration. The number of arousals after falling asleep is significantly decreased in patients on chronic BDZ therapy (cited in [6, 10]).

### 3.7. Lamotrigine (LTG)

Inhibition of presynaptic voltage-sensitive sodium channels and impairment of glutamate release are likely the main antiepileptic mechanisms of lamotrigine [24]. Drowsiness is one of the most common side effects and is dose dependent. Hirsch et al. equated drowsiness with psychomotor slowing, fatigue, or lethargy. The authors observed drowsiness requiring a dose change in 5.7% of patients (*n* = 811; *P* < 0.0001). The same group observed nondose-dependent insomnia in 1.1% (*P* < 0.6449) of patients that lead to dose adjustment [25]. A higher insomnia rate of 6.4% was reported by Sadler [26]. Foldvary et al. investigated an effect of add-on LTG on sleep perception, sleep architecture, and daytime alertness in 10 patients taking either phenytoin (PHT) or carbamazepine (CBZ). Polysomnography demonstrated a statistically significant increase in N2 sleep stage and reduction in N3 sleep stage. A nonstatistically significant increase in REM duration was reported [5]. The majority of subjects experienced decreased sleep latency and improved consolidation of nocturnal sleep. One patient reported more frequent daytime napping. Varying data were reported by Placidi et al. who performed polysomnography in 13 pharmacologically-resistant patients treated with LTG. There were a significant prolongation of REM sleep and a nonsignificant decrease in N3 sleep stage duration in these patients [24]. 

### 3.8. Zonisamide (ZNS)

The antiepileptic effects of zonisamide are mediated through antagonism of voltage-dependent T-type calcium channels, inhibition of voltage-sensitive sodium channels, glutamate blockade, induction of *γ*-aminobutyric acid (GABA) release, and inhibition of carbonic anhydrase. Brodie et al. randomized 351 patients to receive an adjunct seizure treatment of zonisamide at escalating doses or a placebo treatment. Somnolence was reported in 5.4% of patients within the 100 mg/day dosing group, 3.6% of patients within the 300 mg/day group, and 14.4% of patients within the 500 mg/day group [27]. Romigi et al. evaluated the effects of ZNS on nocturnal sleep and daytime vigilance in 12 patients with localization-related epilepsy. Patients were evaluated with PSG, MSLT, the Pittsburgh Sleep Quality Index (PSQI), and the Epworth Sleepiness Scale (ESS) prior to and following 3 months of ZNS treatment. There was no statistical significance detected for any of the assessed measures upon completion of the monitoring period [4].

### 3.9. Levetiracetam (LEV)

The mechanism of action responsible for the antiepileptic effects of levetiracetam is likely modulated by binding to synaptic vesicle protein 2A (SV2A), a transmembrane protein involved in calcium-dependent presynaptic neurotransmitter release [28]. In clinical trials somnolence has been reported in 9.4% of patients taking 1,000 mg/d as an add-on therapy (difference from placebo was 5%) [29], in 11.3% of patients taking 2,000 mg/d as an add-on therapy (difference from placebo was 7%) [29] and in 6.1% of patients taking 3,000 mg/d as an add-on therapy (*P* < 0.584; difference from placebo was 2.3%) [30]. Another trial reported 20.4% of patients complaining of daytime sleepiness while taking 1,000 mg/d as an add-on therapy (difference from placebo was 6.7%) as compared to 18.8% of patients taking 3,000 mg/d as an add-on therapy (difference from placebo was 5.1%) [31]. Tsai et al. evaluated efficacy and safety of levetiracetam therapy in 47 Taiwanese patients. Somnolence was the most commonly reported adverse event: 40.4% in the experimental group and 14.9% in the placebo group. The degree of somnolence was mild in >80% of reported instances. The authors attributed the overall higher incidence of somnolence in the study to the greater degree of concomitant antiepileptic therapy that was reported in the United States and European studies [32]. Cicolin et al. obtained polysomnography and Multiple Sleep Latency Tests in 14 healthy adults treated with ≤2,000 mg/day of levetiracetam. There was a significant increase in time spent in all NREM stages with a relative decrease in time spent in REM sleep. Overall, LEV demonstrated a propensity for sleep consolidation without detrimental effect on daytime vigilance [33]. Bell et al. followed 16 patients with a history of partial epilepsy on stable carbamazepine monotherapy and 12 volunteers after administration of a single dose of 1000 mg of levetiracetam or placebo. The significant findings were an increase in total time spent in stages 2 and 4 (in patients only) of sleep and prolongation of REM latency (in volunteers only). Patient subjects reported having more restful sleep while volunteers were less alert and groggier on waking [34]. A questionnaire-based survey of 288 patients on chronic LEV therapy (90% on polytherapy) revealed an association between sleep problems and the presence of negative behavioral change. In contrast, positive behavioral changes were associated with increased arousal and better subjective cognitive performance [35]. An increase in sleep efficiency was the only significant finding when Cho et al. evaluated 16 patients with partial epilepsy on 1000 mg/day of LEV [23].

### 3.10. Topiramate (TPM)

Topiramate promotes GABA-facilitated inhibition, blocks voltage-dependent sodium channels, modulates voltage-dependent calcium channels, and acts as an antagonist of AMPA receptors [6, 10]. Add-on trials reported a higher frequency of somnolence in patients receiving TPM [36] as compared to gradual titration monotherapy trials [37]. Reife et al. analyzed pooled data from six double-blind, placebo-controlled trials where TPM was used as an adjunctive therapy in 743 adult patients with a diagnosis of localization-related epilepsy. Somnolence was reported in 30% of patients taking 200–400 mg/day and in 28% of patients taking 600–1,000 mg/day. Nearly 90% of central nervous system adverse events were rated as mild or moderate, with the majority occurring shortly after TPM therapy initiation and resolving with continued treatment. Daytime sleepiness was cited as the most common reason for cessation of a TPM regimen, amounting to 3.2% of all discontinuations. Rapid titration was one of the predisposing factors for development of adverse side effects [38]. Glauser summarized data from six clinical trials of topiramate: somnolence was reported by 26.7%, 26.5%, 16.9%, 19.7%, and 27.7% of patients at respective doses of 200, 400, 600, 800, and 1000 mg/day. In trials with adjunctive use of TPM, somnolence was reported in 29% of subjects, whereas, with monotherapy, somnolence was reported in only 13% of patients [36]. When TPM 50 mg/day or 500 mg/day was used as monotherapy in patients with a diagnosis of localization-related epilepsy, somnolence was reported in 14% [39]. An Italian study by Bonanni et al. followed 14 newly diagnosed, pharmacotherapeutically naïve patients with localization-related epilepsy after initiation and titration of up to a 200 mg daily dose of TPM monotherapy for a 15-week period. The amount of daytime sleepiness was measured with the Epworth Sleepiness Scale and the Multiple Sleep Latency Test. Psychomotor performance was evaluated by simple and choice visual reaction times. Patients within the treatment group demonstrated no statistical variation in a daytime vigilance profile or psychomotor performance when compared to similar measures in control subjects [40]. 

### 3.11. Gabapentin (GBP)

Gabapentin is an amino acid that was originally synthesized as a structural analogue of GABA, an inhibitory neurotransmitter in the adult brain. Even though GBP might increase the rate of GABA synthesis in rat brains, the antiepileptic qualities of this AED in humans are likely secondary to glutamate synthesis modulation and inhibition of voltage-sensitive calcium channels [10]. Subjective somnolence has been recorded in 12.1% of patients with epilepsy on GBP therapy [19]. Foldvary-Schaefer et al. investigated the effects of 1,800 mg/day of GBP on sleep architecture and daytime vigilance in ten healthy adult volunteers. The only significant finding reported by the authors was an increase in baseline SWS in the treatment group. When compared to the control group, results were not statistically significant [9]. Placidi et al. evaluated ten patients diagnosed with partial epilepsy after 3 months of stable treatment with 1,800 mg/day of GBP. Polysomnography demonstrated significantly increased REM sleep percentage, prolonged REM mean duration, a decreased number of awakenings, and reduced duration of the N1 stage of sleep [6]. An independent study by Placidi et al. evaluated 18 patients with refractory partial seizures undergoing 4 months of stable treatment with 1800 to 2400 mg/day of GBP. There was a significant increase in REM and SWS percentage, a reduction in the number of awakenings, and a decrease in the length of stage 1 sleep. Sixteen patients reported improvement in sleep quality. There were no significant changes in frequency of interictal epileptiform discharges as evidenced by EEG recording [41].

### 3.12. Pregabalin (PGB)

Pregabalin binds to central nervous system voltage-gated calcium channels demonstrating analgesic, antiepileptic, and anxiolytic effects. Somnolence was reported in 30.1% (versus 12.2% in placebo group) of patients taking 600 mg/day divided into two doses, in 23.4% (versus 12.2% in placebo group) of patients taking 600 mg/day divided into three doses [42], and in 6.1% to 17.4% of patients taking 150 mg/day [43, 44]. This adverse effect seems to be mild or moderate in intensity, occurring within the first two weeks of therapy initiation [45]. When given to 24 healthy volunteers, pregabalin increased time spent in SWS, decreased the number of awakenings, increased total sleep time, reduced sleep-onset latency, and improved sleep efficiency [46]. A randomized, placebo-controlled study of 17 patients with well-controlled partial seizures utilizing polysomnography and subjective sleep questionnaires to explore the effects of 300 mg/day of PGB was performed by de Haas et al. The results demonstrated subjective improvement in the number of awakenings but failed to reveal a statistical significance when analyzed with polysomnography [47]. Romigi et al. compared results of polysomnography and the Epworth Sleepiness Scale in 12 patients with a history of medically refractory seizures before and after a 3-month add-on treatment period with PGB. The authors reported a significant increase in the REM phase and decrease in stage 2 sleep percentage [48]. 

### 3.13. Vigabatrin (VGB)

VGB increases GABA concentration by inhibiting GABA transaminase facilitated GABA uptake [49]. Subjective somnolence has been reported in 11.3% to 25.7% of patients with epilepsy on VGB therapy [19]. Initial reports indicated no changes in polysomnographic and daytime somnolence measures with add-on VGB therapy [10]. Siegel et al. showed prolongation of REM sleep latency with vigabatrin use in three epilepsy patients [50].

### 3.14. Perampanel (PPN)

The proposed antiepileptic effects of perampanel are exerted via noncompetitive antagonism of the AMPA- (*α*-amino-3-hydroxy-5-methyl-4-isoxazole propionic acid-) type glutamate receptor [51]. Subjective somnolence varies depending on the administered dose regimen; it has been reported in 12.2% of patients on 2 mg/day, 5.9% to 20% in patients on 4 mg/day, 12.4% to 26.7% in patients on 8 mg/day, and 17.2% to 19.7% in patients on 12 mg/day [52–57]. 

### 3.15. Felbamate (FBM)

FBM acts on voltage-sensitive calcium channels, NMDA receptors, and, to a milder degree, voltage-sensitive sodium channels [49]. Grosso et al. evaluated 53 children under the age of 4 years on FBM therapy. Somnolence was reported in 13% of patients; an additional 9% of patients had unspecified sleep disturbance complaints [58]. In some epileptic patients felbamate precipitated insomnia with acute and chronic therapy [10]. 

### 3.16. Tiagabine (TGB)

TGB inhibits GABA reuptake, leading to increased concentrations of the neurotransmitter [49]. Subjective somnolence has been reported in 1.1% of epilepsy patients on TGB therapy [19]. TGB has been reported to increase the duration of SWS [19].

### 3.17. Lacosamide (LCM)

LCM exhibits its antiepileptic properties through selective slow inactivation of voltage-gated sodium channels [59]. Subjective somnolence has been reported in 5.1% (for the IV formulation) to 9.7% of patients with epilepsy on LCS therapy [19].

### 3.18. Oxcarbazepine (OXC)

The primary antiepileptic mechanisms of OXC are via voltage-sensitive sodium and calcium channel inhibition [49]. Subjective somnolence has been reported in 15.7% of patients with epilepsy on OXC therapy [19].

## 4. Chronoepileptology

The emerging field of chronoepileptology aims to explore the relationship between seizures and circadian rhythms, with an ultimate goal of utilizing AEDs or neurostimulation in targeted, specific patient populations or individual patients suffering from epileptic seizures in order to maximize their therapeutic effects and minimize adverse events [60]. Circadian rhythms constrained by a 24-hour cycle, under the influence of ambient time clues such as daylight and darkness at night, are an ubiquitous feature of most organisms. The pacemaker underlying the cyclical nature of physiologic, metabolic, and behavior changes is located in the anterior hypothalamic suprachiasmatic nuclei (SCN) in mammals [61, 62]. The circadian system determines the onset of the sleep cycle as well as the shifting between and duration of different sleep stages. Projections from the SCN affect the thalamic and limbic systems—to name a few—networks implicated in epileptogenesis and propagation of epileptic discharges. 

A fluctuation of neurotransmitter and hormone concentrations throughout the sleep-awake cycle has been shown to correlate with a propensity towards epileptic activity. An increasing concentration of adenosine during wakefulness exhibits anticonvulsant properties [61]. Serotonin receptor agonists increase seizure threshold [63]. A nocturnal melatonin peak produced by the pineal body under direct influence of the SCN may have both anti- and proconvulsant effects depending on the administered dose [64–66]. A rising cortisol concentration before awakening is presumed to be protective against seizures [61]. 

A predilection of a particular type of seizure for a specific portion of the sleep cycle has been described. Seizures that are features of idiopathic generalized epilepsies are more likely to occur during drowsiness, slow wave sleep, and awakening from slow wave sleep in animal models [67]. A study of patients with temporal and frontal lobe seizures by Hofstra et al. used salivary levels of melatonin to determine the timing of seizures in relation to circadian rhythms. The peak frequency of temporal lobe seizures occurred within the 6 hours prior to the daily melatonin concentration elevation whereas frontal lobe seizures were most frequent in the 6 to 12 hours after the melatonin concentration increase [62]. 

Intentional changes in administration schedule of AEDs to accommodate circadian rhythms have been reported in patients with epilepsy. Hofstra et al. evaluated 208 patients with an established diagnosis of epilepsy utilizing the Morningness-Eveningness Questionnaire (MEQ). Patients were subdivided into morning, evening, and intermediate circadian types. All three groups demonstrated adaptation of antiepileptic therapy administration to their circadian rhythm with significant delay of administration on workdays compared to free days [68]. This finding illustrates both the tendency to adjust to the work schedule by the patients and increasing probability of breakthrough seizures on off-work days in the light of significant delay of AED administration. A tailored increase of pharmacological therapy in pediatric patients with nocturnal and early-morning seizure leads to higher rates of seizure freedom or spell frequency reduction in a study by Guilhoto et al. [69]. The presence of increased CNS responsiveness to pharmacologic agents during particular phases of the sleep-awake cycle has been proposed as a mechanism responsible for improved seizure control. 

## 5. Conclusion

The influence of seizures on sleep has been well established [12, 70–73]. Although AEDs are only partly responsible for sleep cycle disturbance, an understanding of their impact can help clinicians tailor regimens that prevent unnecessary worsening of sleep quality. Other factors play significant roles in the reciprocal relationship between epilepsy and sleep. The risk factors predisposing to worsening sleep disturbances in adults with epilepsy include the use of older generation AEDs, nocturnal seizures, poor seizure control, comorbid affective disorder, and underlying sleep disorder. In children, factors contributing to a higher likelihood of sleep disturbances are younger age, developmental delay, increased frequency of seizures, a diagnosis of symptomatic epilepsy, polypharmacy, and coexisting sleep disorder. Other important factors that influence the sleep cycle are the rate of AED dose escalation and duration of AED therapy. Newer generation AEDs generally tend to cause less disturbance of the sleep cycle and greater stabilization of sleep architecture. In addition, prolongation of REM sleep and improved sleep efficiency are potential benefits of some newer AEDs that may secondarily contribute to suppression of seizures and decreased epileptogenicity. The influence of circadian rhythms on epileptogenicity is a concept that has been revisited in recent years. A more profound understanding of the relationship between biological rhythmicity throughout the 24-hour awake-sleep cycle and the propensity to have epileptic seizures at particular times within the cycle will allow more directed application of timed AED therapy, personalized VNS parameter settings, changes in sleep hygiene, and/or hormone therapy.

## Figures and Tables

**Table 1:** Influence of anticonvulsant medications on sleep cycle architecture.

	Sleep latency	Arousals	Stage 2/N2	Slow wave sleep	Rapid eye movement sleep	Sleep efficiency
BDZ	↓	↓	↑	↓	↓	↑
BRB	↓	↑	↑	↓	↓	−
PBT	↓	↓	↑	−	↓	↑ or ↓
PHT (acute)	↓	↑	↓	↑	−	
PHT (chronic)	↓	↓	↓	↑ or − or ↓	− or ↓	↓
CBZ (single dose)	↓		↓	↑ or −	↓	↑ or ↓
CBZ (chronic)	↓	↓	−	−	− or ↓	− or ↓
VPA	−	↑	↑	↑ or −	− or ↓	−
FBM						↓
LTG+	−	↓	↑	↓	↑	↑
LEV			↑	↑	↓	↑
ZNS+	−	−	−	−	−	−
TPM						
GBP	−	−	−	↑	−	−
GBP+	−	↓	↓	↑	↑	↑
PGB	↓	↓	↓	↑	−	↑
TGB	−		−	↑	−	↑
ETX		↑	↑	↓	↑	↓
VGB	−	−			− or ↓	
LCM						
OXC						
PPN						

BDZ: benzodiazepine, BRB: barbiturate, PBT: phenobarbital, PHT: phenytoin, CBZ: carbamazepine, VPA: valproate, FBM: felbamate, LTG: lamotrigine, LEV: levetiracetam, ZNS: zonisamide, TPM: topiramate, GBP: gabapentin, PGB: pregabalin, TGB: tiagabine, ETX: ethosuximide, VGB: vigabatrin, LCM: lacosamide, OXC: oxcarbazepine, and PPN: perampanel; +: add-on therapy; −: no changes; empty square: no data available.
